# Catalytical Properties of Free and Immobilized *Aspergillus niger* Tannase

**DOI:** 10.4061/2011/768183

**Published:** 2011-09-12

**Authors:** Abril Flores-Maltos, Luis V. Rodríguez-Durán, Jacqueline Renovato, Juan C. Contreras, Raúl Rodríguez, Cristóbal N. Aguilar

**Affiliations:** Department of Food Science and Technology, School of Chemistry, Autonomous University of Coahuila, Boulevard V. Carranza and González Lobo s/n, 25280 Saltillo, COAH, Mexico

## Abstract

A fungal tannase was produced, recovered, and immobilized by entrapment in calcium alginate beads. Catalytical properties of the immobilized enzyme were compared with those of the free one. Tannase was produced intracellularly by the xerophilic fungus *Aspergillus niger* GH1 in a submerged fermentation system. Enzyme was recovered by cell disruption and the crude extract was partially purified. The catalytical properties of free and immobilized tannase were evaluated using tannic acid and methyl gallate as substrates. *K*_*M*_ and *V*_max_ values for free enzyme were very similar for both substrates. But, after immobilization, *K*_*M*_ and *V*_max_ values increased drastically using tannic acid as substrate. These results indicated that immobilized tannase is a better biocatalyst than free enzyme for applications on liquid systems with high tannin content, such as bioremediation of tannery or olive-mill wastewater.

## 1. Introduction

Tannin acyl hydrolase (TAH) also known as tannase is an enzyme (EC 3.1.1.20) that catalyzes the hydrolysis of ester bonds present in gallotannins, complex tannins, and gallic acid esters [1, 2]. The major applications of tannase are in the elaboration of instantaneous tea and acorn liquor, as well as in the production of gallic acid from tannin-rich agrowastes [3, 4]. Tannase is also utilized as clarifying agent in wine, beer, fruit juices, and coffee-flavored soft drinks [5, 6]. Moreover, it has been proposed the use of this enzyme for bioremediation of effluents from tanneries and to improve the nutritional properties of tannin-rich forage [7]. 

Despite the several important applications of tannase in food, feed, chemical and pharmaceutical industries, high scale use of this enzyme is severally restricted due to high production costs. Thus tannase is considered a specialty enzyme. Therefore, there is a continuous search for new sources of tannase [8–10], as well as improved methods for production, recovery, and application of the enzyme [11–13].

In order to overcome some limitations of free tannase, several attempts have been made to immobilize the enzyme on a suitable matrix [14–24]. Enzyme immobilization facilitates the efficient recovery and reuse of costly enzymes and enables their use in continuous, fixed-bed operation. Immobilized enzymes are more easy to handle and to separate from the product, thereby minimizing or eliminating protein contamination of the product. Additionally, immobilized enzymes are often more stable than the free ones, allowing the repeated reuse of the biocatalyst [25]. 

One of the most convenient methods for enzyme immobilization is the entrapment in Ca-alginate beads. The major advantage of this technique is the simplicity by which spherical beads can be obtained by dripping a polymer-cell suspension into a medium containing positively charged ions. The particles formed are transparent, mechanically stable, nontoxic, and cheap [26, 27]. 

We previously reported the production, and purification of a novel tannase from the xerophilic fungus *Aspergillus niger *GH1 [28]. This enzyme showed interesting properties such as good activity and stability at high temperature, low inhibition by metal ions and other additives and considerable stability at a wide range of pH. Therefore, in the present study, tannase from *A. niger* GH1 was immobilized in Ca alginate beads and the catalytical properties of immobilized tannase were compared with those of free enzyme.

## 2. Materials and Methods

### 2.1. Microorganism and Culture Conditions

*Aspergillus niger* GH1, utilized for tannase production, was obtained from the UAdeC-DIA culture collection. This strain was previously isolated from Mexican semidesert and characterized as tannase producer [29]. Fungal spores were stored at −40°C in a cryoprotectant medium composed of glycerol and skim milk.

Microorganism was propagated by transferring conserved spores to Erlenmeyer flasks with Czapek-tannic acid agar and incubating at 30°C for 4–6 days. After this, spores were harvested with Tween 80 (volume ratio of 0.01%) and counted in a modified Neubauer chamber before inoculation.

Tannase was produced by *A. niger *in submerged culture. The culture medium for tannase contained (g/L) KH_2_PO_4_: 2.19; (NH_4_)_2_SO_4_: 4.38; MgSO_4_·7H_2_O: 0.44; CaCl_2_·7H_2_O: 0.044; MnCl_2_·6H_2_O: 0.009; NaMoO_4_·2H_2_O: 0.004; FeSO_4_·7H_2_O: 0.06. Salt solution was autoclaved at 12°C for 15 min and cooled at room temperature. Tannic acid was added to the salt solution to a final concentration of 12.5 g/L, then the pH was adjusted at 5.5 with 1 N NaOH, and then the culture media was sterilized again by filtration through 0.45 *μ*m nylon membranes. Tannase production was carried out in 1 L Erlenmeyer flasks containing 250 mL of culture medium inoculated with 1 × 10^7^ *A. niger* spores/mL. Flasks were incubated for 24 h at 30°C with constant agitation at 250 rpm.

### 2.2. Tannase Recovery

Crude enzymatic extracts from SmF were obtained by filtering the biomass through Whatman no. 41 filter paper. Mycelial cells retained were washed with physiological solution, frozen with liquid nitrogen and milled in a mortar. The macerate was recovered with acetate buffer (100 mM, pH = 5.5). Crude extract was put into a 10 kD MWCO cellulose membrane (Sigma, St. Louis, USA) and dialyzed against water. Dialyzed extract was concentrated (12.5-fold) with polyethylene glycol-6000 as described by Sharma and coworkers [30]. Concentrated extract was applied into a HiTrap G25 column, eluted with citrate buffer (100 mM, pH 5.0) and fractionated in an AKTA prime FPLC system (Amersham, Piscataway, USA).

### 2.3. Enzyme Immobilization

Tannase from *A. niger* was immobilized in Ca alginate beads. 4 mL of partially purified tannase (288 U/L) were mixed with 46 mL of 2.0% sodium alginate solution to get homogeneity. Then the mixture was added with constant agitation and a temperature of 4°C to 0.6 M CaCl_2_ solution as droplets using a glass burette. The beads were kept in 0.1 M CaCl_2_ at 4°C for about 2 h and then washed briefly with sterile water.

### 2.4. Kinetic Constants of Tannase

Values of *K*_*M*_ and *V*_max_ were calculated for free and immobilized tannase using methyl gallate and tannic acid as substrates in citrate buffer (50 mM, pH 5.5). Hydrolysis was carried out in a 250 mL glass jacketed bioreactor. 50 beads of immobilized tannase or the equivalent in free enzyme was added to 100 mL of substrate with constant agitation and controlled temperature at 30°C, and 1 mL samples were withdrawn at regular intervals. Reaction was stopped with 0.2 mL of 2 N HCl, and the reaction mixture was filtered through a 45 *μ*m membrane and analyzed for gallic acid by an HPLC procedure [31]. Tannase activity rate was estimated as V (IU) following the Michaelis-Menten equation. Estimation of different parameters for the equation was obtained through the linearization method of Lineweaver-Burk, using the Solver utility of the program Excel (Microsoft, Redmond, USA).

### 2.5. Analytical Methods

Tannase activity was assayed using HPLC methodology, essentially as described by Beverini and Metche [31] with slight modifications. In brief, the enzyme (50 *μ*L) was added to 1 mL of methyl gallate 3 mM. The reaction mixture was incubated at 30°C for 30 min. The reaction was stopped with 2M HCl. Each sample was filtered through a 45 *μ*m membrane prior to HPLC analysis. One unit of enzyme (IU) was defined as the amount of enzyme able to release 1 *μ*mol gallic acid per minute of culture filtered under the standard assay conditions. Protein estimation was done as described by Bradford [32].

## 3. Results and Discussion

### 3.1. Tannase Production and Recovery

Tannase production was carried out by *A. niger* GH1 in submerged fermentation. Under the described conditions, tannase was expressed mainly intracellularly (data not shown). At the end of incubation, 1.16 g of biomass were obtained from 2 L of culture broth. After cell disruption it were recovered 200 mL of a crude extract with 15.68 IU of tannase per liter and a specific activity of 7.29 IU per mg of protein. 

Tannase production was significantly lower than previously reported by our group [28]. This may be related with the culture system utilized. In that case, Mata-Gómez and coworkers [28] utilized a tannase produced by *A. niger *GH1 in a solid-state fermentation system. The enzyme was produced mainly extracellularly, and the volumetric activity reached about 400 IU/L. Lekha and Lonsane [33] reported that in submerged fermentation, tannase production by *A. niger* is intracellular during the first 48 h and the enzyme is subsequently excreted. In contrast, tannase production in solid-state fermentation is completely extracellular. 

On the other hand Barthomeuf and coworkers found that, during the first hours of submerged fermentation, tannase remains strongly bound to the mycelium of *Aspergillus niger* and no more than 5% of the enzyme may be removed by physical methods, such as cell disruption [34]. However, at industrial level tannase is produced mainly by submerged fermentation due to more standardized methodology and equipment. 

Crude extract was subjected to a recovery process consisting of dialysis, concentration, and gel filtration chromatography. This protocol led to 5.4-fold purification and 5.6-fold concentration with a recovery yield higher than 90%.

We previously reported the purification of an extracellular tannase from *A. niger.* In that case, through a protocol consisting of ultrafiltration, ionic exchange, and gel filtration chromatography, a 42-fold purification was obtained with 0.3% yield [28]. In the present paper, we concentrated and partially purified the enzyme according with the needs of an enzyme for industrial use. Industrial enzymes require a certain degree of purity to avoid undesirable reactions and the presence of proteins and other foreign compounds in the final product. The degree of purity required is lower for immobilized enzymes due to entrapment of the biocatalyst [25]. 

Most of the reported protocols for tannase involve several precipitation and chromatographic steps. For example, Mahendran and coworkers purified a *Paecilomyces variotii *tannase by a treatment with activated charcoal followed by precipitation with ammonium sulfate, ionic exchange chromatography, and gel filtration. They obtained a 30.5-fold purification of the enzyme, but with a recovery yield of 17.6% [20]. On the other hand, Sharma and coworkers purified a *Penicillium variable* tannase through a two-step procedure. They utilized ultrafiltration and gel filtration and obtained a 135-fold purification with a recovery yield of 91% [21]. 

### 3.2. Enzyme Immobilization

Tannase was efficiently immobilized in Ca alginate beads. During immobilization, it was obtained 253 beads of 3.5 mm of diameter per mL of enzyme extract. Since no residual activity or protein was detected in CaCl_2_ solution or in the wash water, it was assumed that the enzyme was completely trapped.

Recently, Schons and coworkers reported the immobilization of a *Paecilomyces variotii *tannase by entrapment into several polysaccharide matrixes [35]. Besides the fact that the best encapsulation efficiency (57%) was obtained with pectin, tannase immobilized in alginate beads (15% of encapsulation efficiency) was more efficient for hydrolysis of tannic acid. 

### 3.3. Kinetic Constants of Tannase

**Figure 1:**
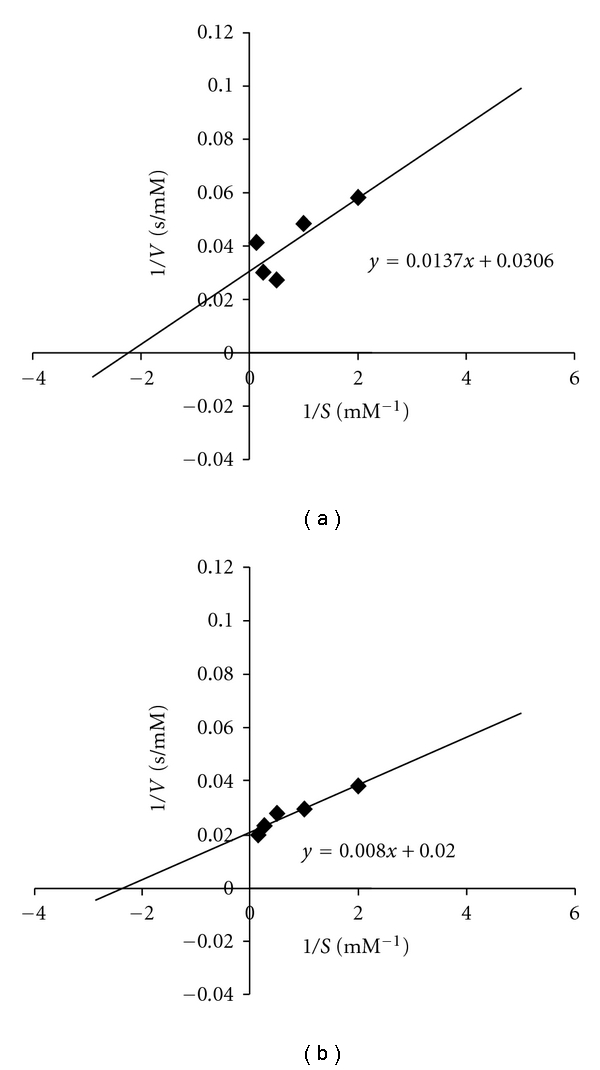
Lineweaver-Burk plots for free tannase using methyl gallate (a) and tannic acid (b) as substrate.

**Figure 2:**
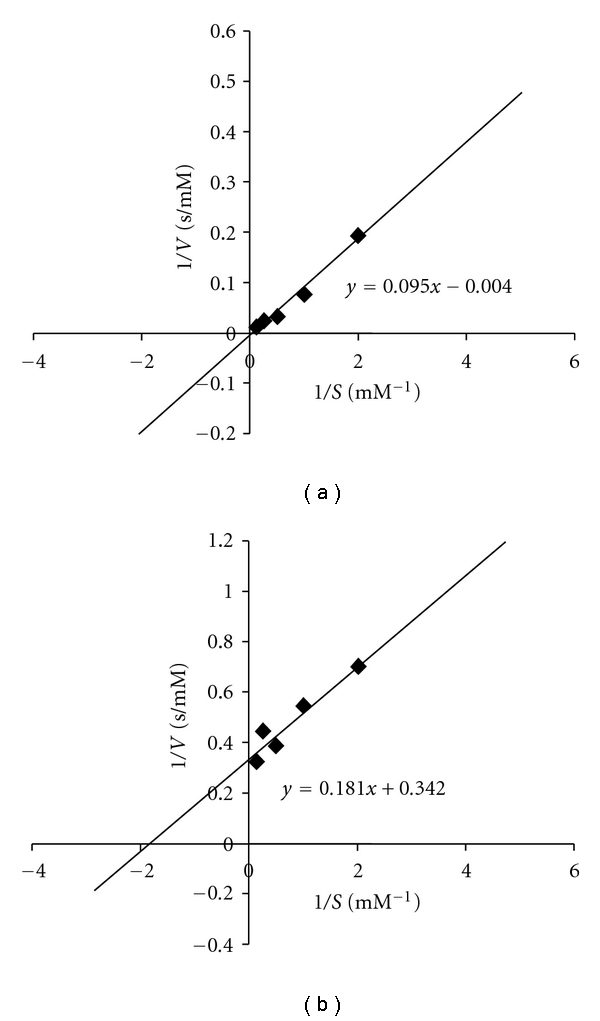
Lineweaver-Burk plots for immobilized tannase using methyl gallate (a) and tannic acid (b) as substrate.

Kinetic parameters of free and immobilized were evaluated at 30°C and a pH of 5.5 using tannic acid and methyl gallate as substrate. *K*_*M*_ and *V*_max_ were obtained with the linearization method of Lineweaver-Burk. The kinetic constants are summarized in Table 1, Lineweaver-Burk plots are showed in Figures 1 and 2. 

*K*_*M*_  values for free enzyme using methyl gallate and tannic acid as substrate are very close among them (0.433 and 0.400 mM, resp.). When tannase was immobilized, these *K*_*M*_ values were notoriously increased (1.3- and 59-fold for methyl gallate and tannic acid, resp.). 

These results indicate that *A. niger *GH1 tannase produced in submerged fermentation has almost the same affinity for methyl gallate and tannic acid. In contrast, the tannase produced by this microorganism in solid-state fermentation has more affinity for tannic acid than for methyl gallate [28]. These catalytical disparities among the tannases could be related to differences in glycosylation among the enzyme produced in solid-state and submerged fermentation, as suggested by Renovato and coworkers [36].

The *K*_*M*_ value of tannase increased significantly after immobilization in Ca alginate, especially when tannic acid was used as substrate. This is a common phenomenon during enzyme immobilization [14, 18, 22, 24]. The increase of *K*_*M*_ is partially due to mass transfer resistance of the substrate into the polymer matrix, and mass transfer resistance appears to be drastic in substrates such as tannins [14]. For example, Yu and coworkers [24] immobilized an *A. niger* tannase with a coacervate calcium alginate membrane. In that case, the *K*_*M*_ value increased 4 times with immobilization. Similar results were reported by other authors (Table 1). 

Hydrolysable tannins have the ability to complex with macromolecules such as carbohydrates but, to maintain its binding capacity, tannins must have more than two acidic unit constituents esterified to the glucose core [4]. Thus, the interaction between alginate and tannic acid is much higher than that with methyl gallate, as indicated by these results.

On the other hand, the *V*_max_ value for intracellular tannase from *A. niger *is higher using tannic acid as substrate in both free and immobilized forms. In addition, immobilization resulted in an increase in *V*_max _  when tannic acid was used as substrate. Typically, immobilized enzymes have lower *V*_max_ values than their free counterparts [37], and this phenomenon is associated with conformational changes of the enzyme during immobilization [18]. Higher *V*_max_ values after immobilization have been reported for inulinase [38], invertase [39], and *β*-galactosidase [40]. However, this is an unusual behavior and the mechanistic basis for this positive feature requires further investigation [37].

The increase of the maximal velocity of reaction and the *K*_*M*_ value makes the immobilized tannase a better biocatalyst than free enzyme for applications on liquids with high tannin content, such as bioremediation of tannery or olive-mill wastewater. In addition, the immobilization technique allows the recovery and reutilization of the enzyme.

## 4. Conclusions

Tannase was efficiently immobilized in Ca alginate beads. The immobilized enzyme presented interesting catalytical properties, different from the free enzyme. These catalytical differences could be related with mass diffusion coefficients and conformational changes of the protein as well as the interaction of the substrate with the polymer matrix. These properties of the enzyme and the possibility of recovery and reutilization make the immobilized tannase from *Aspergillus niger* GH1 an interesting biocatalyst with potential application in liquid systems with high tannin content, such as bioremediation of tannery or olive-mill wastewater. 

## Figures and Tables

**Table 1:** Kinetic constants of free and immobilized tannase.

Microorganism	Substrate	Free tannase		Immobilized tannase		Reference
		*K*_*M*_ mM	*V*_ Max_ *μ*mol/min	*K*_*M*_ mM	*V*_ Max_ *μ*mol/min	
*Rhizopus oryzae*	Sal seed	30.9*	4.4**	39.9*	4.0**	[18]
*Rhizopus oryzae*	Myrobalan	26.3*	9.4**	29.0*	2.4**	[18]
*Rhizopus oryzae*	Tea leaf	26.4*	0.47**	30.5*	0.46**	[18]
*Aspergillus oryzae*	Tannic acid	7.35	80	11.76	40	[14]
*Aspergillus niger*	Tannic acid	0.3	0.013	0.6	0.020	[24]
*Aspergillus niger*	Tannic acid	1.1 × 10^−5^	416	1.1 × 10^−5^	131	[22]
*Aspergillus niger*	Tannic acid	0.400	0.05	23.75	0.25	This study
*Aspergillus niger*	Methyl gallate	0.433	0.033	0.529	0.003	This study

*These results are expressed as mg/mL.

**These results are expressed as U/mL/h.
